# Digital Patient-Reported Outcome Measures Assessing Health-Related Quality of Life in Skull Base Diseases—Analysis of Feasibility and Pitfalls Two Years after Implementation

**DOI:** 10.3390/healthcare11040472

**Published:** 2023-02-06

**Authors:** Christine Steiert, Johann Lambeck, Tanja Daniela Grauvogel, Juergen Beck, Juergen Grauvogel

**Affiliations:** 1 Department of Neurosurgery, Medical Center—University of Freiburg, Faculty of Medicine, University of Freiburg, 79106 Freiburg, Germany; 2 Department of Neurology and Clinical Neurophysiology, Medical Center—University of Freiburg, Faculty of Medicine, University of Freiburg, 79106 Freiburg, Germany; 3 Department of Otorhinolaryngology—Head and Neck Surgery, Medical Center—University of Freiburg, Faculty of Medicine, University of Freiburg, 79106 Freiburg, Germany

**Keywords:** PROM, digital outcome measures, skull base, neurosurgery, HRQoL, quality of life, patient-centered care

## Abstract

Health-related quality of life (HRQoL) assessment is becoming increasingly important in neurosurgery following the trend toward patient-centered care, especially in the context of skull base diseases. The current study evaluates the systematic assessment of HRQoL using digital patient-reported outcome measures (PROMs) in a tertiary care center specialized in skull base diseases. The methodology and feasibility to conduct digital PROMs using both generic and disease-specific questionnaires were investigated. Infrastructural and patient-specific factors affecting participation and response rates were analyzed. Since August 2020, 158 digital PROMs were implemented in skull base patients presenting for specialized outpatient consultations. Reduced personnel capacity led to significantly fewer PROMs being conducted during the second versus (vs.) the first year after introduction (mean: 0.77 vs. 2.47 per consultation day, *p* = 0.0002). The mean age of patients not completing vs. those completing long-term assessments was significantly higher (59.90 vs. 54.11 years, *p* = 0.0136). Follow-up response rates tended to be increased with recent surgery rather than with the wait-and-scan strategy. Our strategy of conducting digital PROMs appears suitable for assessing HRQoL in skull base diseases. The availability of medical personnel for implementation and supervision was essential. Response rates during follow-up tended to be higher both with younger age and after recent surgery.

## 1. Introduction

During the recent shift from disease-centered to patient-centered medical care, the assessment of health-related quality of life (HRQoL) has become increasingly important [1,2]. In the context of individualized care, patients are involved in medical decision-making processes [3]. “Shared decision making” is applied in surgical disciplines, for example, when different therapeutic options are available that are considered to be equivalent, or during the monitoring of so-called “incidental findings” [4,5]. In the field of neurosurgery, this concept appears to be of central relevance for the management of skull base pathologies. Considering the mainly benign tumors in this region, a wait-and-see strategy must often be weighed against various therapeutic approaches. These individual decision-making processes require not only knowledge about the natural course of the disease and the outcome to be expected with treatment, but also awareness of HRQoL and disease-specific factors in the affected individual [6,7]. Benign skull base tumors comprise about 30% of all intracranial tumors. There are first reports on the assessment of HRQoL using patient-reported outcome measures (PROMs) in several neurosurgical diseases in the recent literature [8,9,10,11,12,13]. Here, evidence has emerged that interviewing people affected by cranial pathologies can be associated with special challenges, for example, due to more frequent cognitive impairment and accompanying mental disorders caused by the neurosurgical disease. Specifically in the context of brain tumors, various preconditions arise depending on their type, dignity, size, and localization. These aspects are essential to consider as PROMs become more established and undergo further development in neurosurgical care [6,14,15]. Reports on the use of non-digital PROMs in benign brain tumors can be found, e.g., in the context of vestibular schwannoma, but descriptions of the application of a digital format are lacking [16,17,18]. In other disciplines, such as orthopedic surgery, first reports on digital PROM assessment can be found [19,20].

The current study aims to evaluate an elaborated strategy of systematic assessment of HRQoL using digital PROMs in skull base diseases in a specialized neurosurgical tertiary care center, with a protocol combining generic and specific questionnaires. The feasibility and pitfalls are determined by analyzing patient-specific factors and infrastructural conditions, including those related to the coronavirus disease 2019 (COVID-19) pandemic, during which digital PROMs were implemented. The evaluation of the protocol to assess digital PROMs is intended to investigate feasibility and to identify pitfalls that require modification, so that, in the future, a suitable digital format will enable the acquisition of valid data that can be included in clinical care. The integration of HRQoL seems particularly important in the context of benign skull base diseases for individual decision making and long-term management. To our knowledge, no comparable approach specifically focusing on the analysis of HRQoL via digital PROMs in neurosurgical skull base patients can be found in the literature.

## 2. Materials and Methods

### 2.1. Study Design

Systematic assessment of HRQoL using digital PROMs (Heartbeat Medical, HRTBT Medical Solutions GmbH, Berlin, Germany) was performed in the Department of Neurosurgery, a tertiary referral center specialized in skull base diseases, since August 2020. The purpose of the current retrospective study is to analyze factors that potentially influence participation in digital PROMs and response rates during follow-up assessments. In this context, the infrastructural aspects (e.g., availability of medical staff, the selection of questionnaires, and the potential impact of the COVID-19 pandemic) and patient-specific factors (e.g., age, type of disease, and clinical procedure) are investigated. The study intends to identify possible difficulties and pitfalls that require optimization to enable routine use of PROMs in the care of skull base diseases. Inclusion criteria were as follows: age ≥ 18 years, presence of a neurosurgical skull base disease, presentation to the outpatient clinic, and consent to participate in digital PROMs. Exclusion criteria were as follows: age < 18 years, presence of other than neurosurgical skull base disease, and lack of consent to participate. Informed consent to data collection and storage was obtained from patients with their participation in digital PROMs by medical staff in the outpatient clinic. The retrospective analysis was approved by the independent ethics committee of our medical center (reference no. 22-1295-S1-retro, 9 August 2022) and is reported according to institutional guidelines.

### 2.2. Implementation of PROMs

Digital PROMs were implemented in 158 adult patients with skull base pathologies during outpatient visits, following a sophisticated protocol, with selection of questionnaires depending on the underlying disease and localization of the process. All questionnaires were administered in their standardized, validated German versions. Participants with insufficient language skills could be supported by accompanying persons or use smartphone tools for translation. Patients presented either for consultation after first diagnosis, for planning of a surgery, or for regular follow-up visits (i.e., check-ups after treatment or monitoring as part of wait-and-scan strategies). The initial PROM assessments were completed during outpatient visits using tablets (the initial assessments are referred to as T1). Persons with visual or other neurological impairment may have had to be assisted by an accompanying person to carry out the assessment. With consent to further follow-up assessments, patients received the same questionnaires automatically by email after 3 and 12 months (referred to as T2 and T3, respectively). In case of non-response, reminder emails were sent automatically by the system at fixed intervals. Patients had the option to unsubscribe from the system via a link included in the follow-up emails. The results of the questionnaires were transferred and stored in the hospital’s internal system with the patients’ electronic medical records.

### 2.3. Disease-Specific Selection of Questionnaires

Prior to implementation of PROMs, the following subgroups were defined for selection of questionnaires according to the underlying disease and localization of the process:

- Subgroup AMSB (anterior and middle skull base):
  patients with (mainly benign) tumors located in the region of the anterior or middle skull base (mostly meningiomas), excluding tumors of the sella.
- Subgroup CPA (cerebellopontine angle):
  patients with (mainly benign) tumors located in the cerebellopontine angle or petroclival region (e.g., meningiomas, (vestibular) schwannomas, epidermoid cysts, etc.).
- Subgroup NVC (neurovascular conflict):
  patients with neurovascular compression syndromes (mostly trigeminal neuralgia).

In all three subgroups (together referred to as “group ALL”), assessment of general HRQoL and comorbidities was conducted with three standardized questionnaires: EQ-5D-5L/EQ-5D-VAS, 15D, and Self-Administered Comorbidity Questionnaire (SCQ). To determine specific problems associated with the underlying disease or its localization, additional questionnaires were administered in subgroups AMSB and CPA. The National Eye Institute Visual Function Questionnaire (NEI-VFQ-25) was implemented in subgroup AMSB to evaluate the influence of visual dysfunction. To assess the potential impact of hearing impairment, dizziness, and facial nerve dysfunction in subgroup CPA, the Penn Acoustic Neuroma Quality of Life (PANQOL) scale, the Dizziness Handicap Inventory (DHI), the Facial Disability Index (FDI), and the Facial Clinimetric Evaluation (FaCE) were included. In subgroup NVC, the Patient Satisfaction Index (PSI) was added to measure treatment satisfaction.

### 2.4. Statistical Analysis

Methods of descriptive statistics were used. Categorical data are presented as absolute and relative frequencies (in %). For numerical data, either mean values with standard deviation (SD) or median values with minimum/maximum and interquartile range (IQR) were calculated, as required. Statistical differences were evaluated using the *t* test or Mann−Whitney test, as appropriate. The level of significance was set at *p* < 0.05. Statistical analysis was performed using GraphPad Prism software version 9.4.1 for Mac (GraphPad Software, San Diego, CA, USA).

## 3. Results

### 3.1. Availability of PROMs and Participation Rate

**Figure 1:**
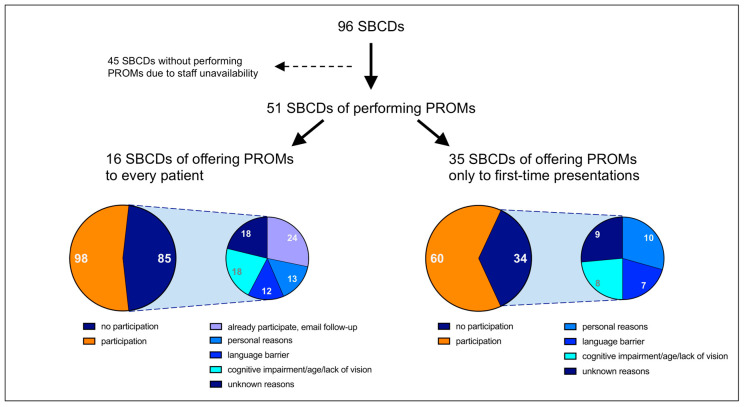
Availability of PROMs within 2 years after implementation, participation rate, and reasons for non-participation. Numbers in white/gray = number of patients (each left circle) who participated (orange) or did not participate (dark blue), or number of patients subdivided according to reasons for non-participation (each right circle).

In the current study, we analyzed the 24 months (August 2020–July 2022) since digital PROMs were implemented in the Department of Neurosurgery to assess HRQoL in patients with skull base diseases. During this period, special consultation hours for skull base diseases were held in our outpatient clinic on 96 days (“skull base consultation day” = SBCD), on which a total of 1120 skull base patients presented (mean: 11.7 patients per SBCD). Conducting PROMs required the capacity of the outpatient medical staff, who had to register patients in the system, instruct and support them in (technical) performance, and manage the hardware. Therefore, the capacity for PROMs was dependent on staff availability; it was not always possible to conduct PROMs, and they were frequently applied with reduced capacity. Overall, PROMs were performed on 51 SBCDs during the analysis period, while the offer to participate was made to every patient presenting on 16 SBCDs (participation rate of 53.55%) and only to patients presenting for the first time (after initial diagnosis or before/after surgery) on 35 SBCDs (participation rate of 63.83%). Further information is given in Figure 1.

### 3.2. Conduction of PROMs in Relation to the COVID-19 Pandemic

**Figure 2:**
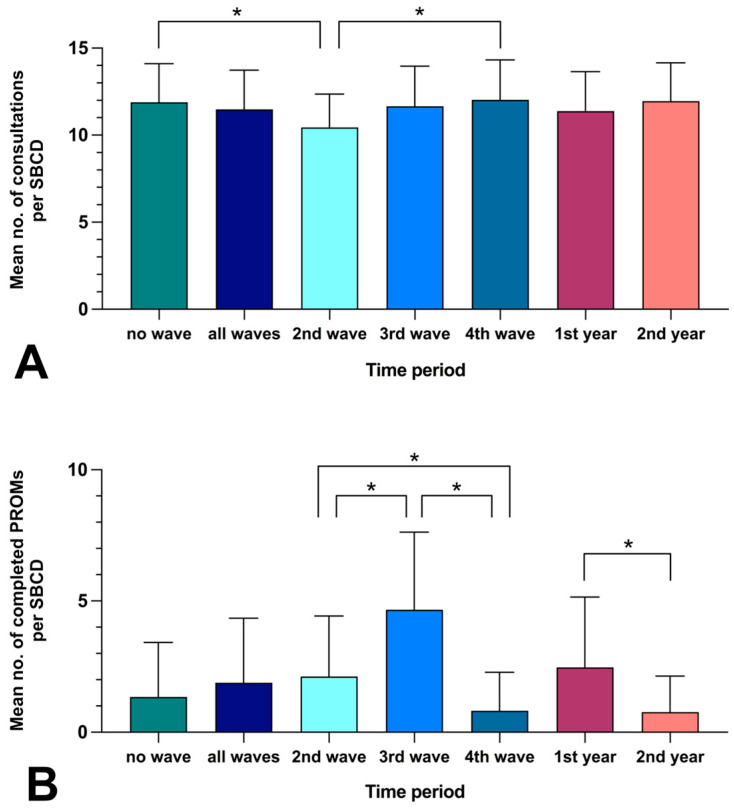
Analysis of the conduction of PROMs in relation to the COVID-19 pandemic. no. = number, * = statistically significant difference (*p* < 0.05), (**A**,**B**): Comparison of the mean number (with standard deviation) of (**A**) consultations per “skull base consultation day” (SBCD) or of (**B**) completed PROMs per SBCD (in relation to the total of 96 SBCDs), illustrated for the 13 months during COVID-19 waves (“all waves” or subdivided into “second wave” (2nd wave), “third wave” (3rd wave), and “fourth wave” (4th wave)) and the 11 months beyond COVID-19 waves (“no wave”), and illustrated for the “first year” (1st year) and “second year “ (2nd year) after implementation of PROMs.

The analysis period included the 2nd, 3rd, and 4th wave of the COVID-19 pandemic. According to the German Federal Statistical Office, the 2nd, 3rd and 4th wave covered a total period of 13 months. During this period, the mean number of patients presenting was 11.48 per SBCD; in the 11 months beyond COVID-19 waves, it was 11.89 per SBCD. The mean number of consultations remained relatively stable during the 24 months, except during the 2nd wave, when it was significantly reduced (see Figure 2). As a result of staff unavailability, PROMs could not be offered during COVID-19 waves on a total of 22 SBCDs (mean: 1.69/month) and beyond COVID-19 waves on a total of 23 SBCDs (mean: 2.09/month). Independent of the pandemic, when comparing the first (August 2020–July 2021) and second (August 2021–July 2022) year after implementation, no PROMs could be offered on 15 SBCDs during the first year (mean: 1.25/month) and on 30 SBCDs during the second year (mean: 2.5/month) due to staff unavailability. The mean number of completed PROMs per SBCD did not differ significantly when comparing the period during and beyond COVID-19 waves (1.89 vs. 1.34 per SBCD, respectively). However, the mean number of completed PROMs per SBCD was significantly reduced during the second year after implementation when compared with the first year (0.77 vs. 2.47 per SBCD, respectively; *p* = 0.0002). Further details are listed in Figure 2.

### 3.3. Response Rate to PROMs Depending on Subgroup and Neurosurgical Procedure

A total of 158 patients (group ALL) completed T1. Of these patients, 83 (52.5%) completed T2, and 61 (38.6%) completed T3. Of the total number of patients who completed T1, 69 (43.7%) belonged to subgroup AMSB and 68 (43.0%) to subgroup CPA; the remaining subgroup NVC comprised 21 (13.3%) patients. Regarding response rates during follow-up, there were no significant differences between subgroups AMSB and CPA. In subgroup AMSB, T2 was completed by 36/69 (52.2%) and T3 by 27/69 (39.1%) patients; in subgroup CPA, T2 was completed by 41/68 (60.3%) and T3 by 26/68 (38.2%) patients. In subgroup NVC, T2 was completed by 6/21 (28.6%) and T3 by 8/21 (38.1%) patients. At the time of analysis, 3 patients had not reached the date for T2, and 27 patients had not reached the date for T3. These pending assessments were equally distributed among the subgroups.

**Figure 3:**
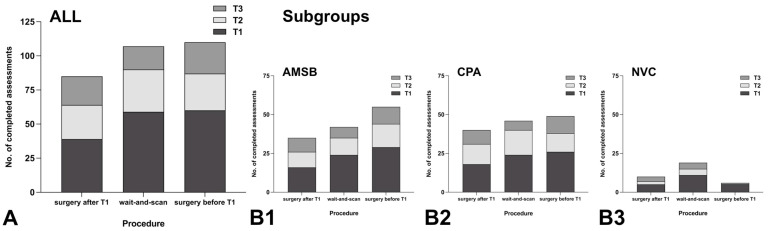
Evaluation of the response rate to PROMs depending on subgroup and neurosurgical procedure. ALL = total group, AMSB = subgroup “anterior and middle skull base”, CPA = subgroup “cerebellopontine angle”, NVC = subgroup “neurovascular conflict”, no. = number, T1 = initial assessment, T2 = follow-up assessment after 3 months, T3 = follow-up assessment after 12 months, “surgery after T1” = surgical treatment was performed after the initial assessment and before the follow-up assessment after 3 months, “wait-and-scan” = wait-and-scan strategy, “surgery before T1” = surgical treatment had already been performed before the initial assessment. (**A**): Number of completed assessments T1, T2, and T3 according to the neurosurgical procedure among ALL. (**B**): Number of completed assessments T1, T2, and T3 according to the neurosurgical procedure among AMSB (**B1**), CPA (**B2**), and NVC (**B3**).

Of the 158 patients who had completed T1, 39 (24.7%) underwent surgery between T1 and T2, 59 (37.3%) followed a wait-and-scan strategy, and 60 (38.0%) had already undergone surgery before T1 (19 within the last 6 months). The response rate during follow-up was highest in patients who underwent surgery between T1 and T2, where 25/39 (64.1%) completed T2 and 21/39 (53.8%) completed T3. In patients following a wait-and-scan strategy, 31/59 (52.5%) completed T2 and 17/59 (28.8%) completed T3. In patients who had already undergone surgery before T1, 27/60 (45.0%) completed T2 and 23/60 (38.3%) completed T3. Further details are presented in Table 1 and in Figure 3.

### 3.4. Response Rate to PROMs Depending on Age

**Figure 4:**
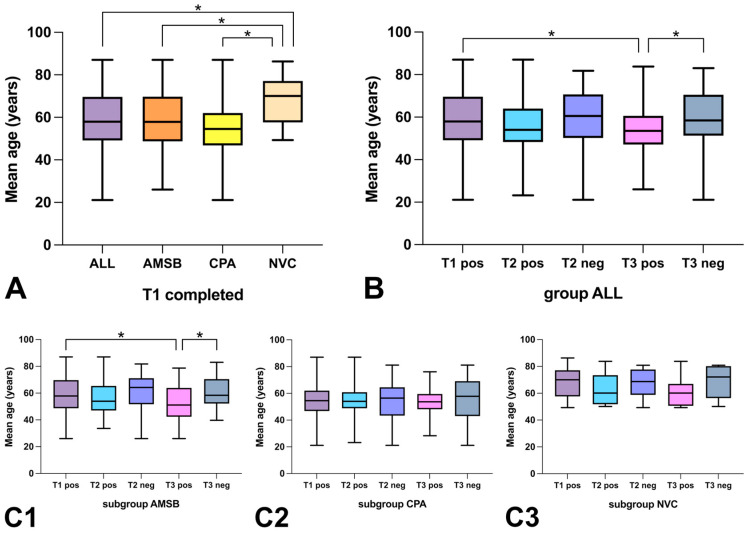
Evaluation of the response rate to PROMs depending on age. (group) ALL = total group, (subgroup) AMSB = subgroup “anterior and middle skull base”, (subgroup) CPA = subgroup “cerebellopontine angle”, (subgroup) NVC = subgroup “neurovascular conflict”, T1 completed/T1 pos = completed initial assessments T1, T2/T3 pos = completed follow-up assessments T2/T3, T2/T3 neg = not-completed follow-up assessments T2/T3, * = statistically significant difference (*p* < 0.05). (**A**): Comparison of the mean age of the total group and the subgroups AMSB, CPA, and NVC at initial participation (T1), presented as median values (black horizontal lines) with 25–75% percentiles (colored box) and minimum/maximum (black vertical lines). (**B**,**C**): Comparison of the mean age of participants who completed T1 (T1 pos), T2 (T2 pos), and T3 (T3 pos) and those who did not complete T2 (T2 neg) or T3 (T3 neg) (values presented the same as in “(**A**)”) according to the total group (**B**) or the subgroups AMSB (**C1**), CPA (**C2**), or NVC (**C3**).

The mean age of group ALL at T1 was 58.39 years (y), and those of subgroups AMSB and CPA was 58.67 y and 55.23 y, respectively. The mean age of subgroup NVC was significantly higher at 67.71 y, compared with both group ALL (*p* = 0.0033) and subgroups AMSB (*p* = 0.0071) and CPA (*p* = 0.0002) (see Figure 4A). Both in group ALL and in subgroup AMSB, the mean age of patients who completed T3 (T3 pos) was significantly lower compared with the baseline (T1 pos) (group ALL: 54.11 vs. 58.39 y (*p* = 0.0322) and subgroup AMSB: 52.19 vs. 58.67 y (*p* = 0.0346)) and compared with those not completing T3 (T3 neg) (group ALL: 54.11 vs. 59.90 y (*p* = 0.0136) and subgroup AMSB: 52.19 vs. 62.09 y (*p* = 0.0023)). The mean age according to completion of follow-up assessments is given in Figure 4B,C.

### 3.5. Response Rate to PROMs Depending on Gender

**Figure 5:**
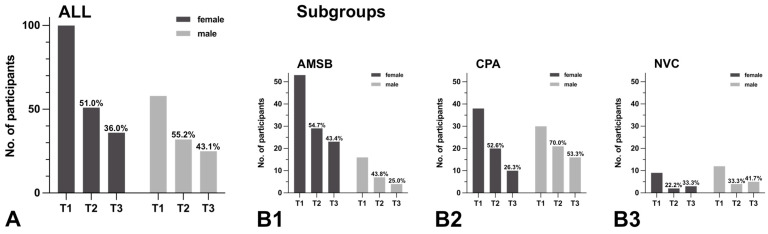
Evaluation of the response rate to PROMs depending on gender. ALL = total group, AMSB = subgroup “anterior and middle skull base”, CPA = subgroup “cerebellopontine angle”, NVC = subgroup “neurovascular conflict”, no. = number, T1 = initial assessment, T2 = follow-up assessment after 3 months, T3 = follow-up assessment after 12 months. (**A**,**B**): Number of completed assessments T1, T2, and T3 in relation to gender among ALL (**A**) and the subgroups AMSB (**B1**), CPA (**B2**), or NVC (**B3**) (the proportion of completed T2/T3 assessments in relation to the corresponding number of T1 assessments, sorted by gender, is presented as a percentage).

Among 158 patients, 100 (63.3%) were female and 58 (36.7%) were male. For females, response rates during follow-up were 51/100 (51.0%) for T2 and 36/100 (36.0%) for T3. For males, response rates were 32/58 (55.2%) for T2 and 25/58 (43.1%) for T3. In subgroup AMSB, 53/69 (76.8%) were female and 16/69 (23.2%) were male; in contrast, subgroup CPA was more balanced, with 38/68 (55.9%) females and 30/68 (44.1%) males. In subgroup AMSB, higher response rates for T2 and T3 were observed among females, whereas in subgroup CPA, higher response rates for T2 and T3 were observed among males. Detailed information is presented in Figure 5.

## 4. Discussion

The trend toward patient-centered medical care implies an increasing impact of factors, such as symptom burden, functional status, and HRQoL on individual treatment decisions [21,22,23]. These parameters can be systematically assessed in routine clinical practice through the use of PROMs, with digital formats providing increased accessibility and simplification of data collection [4,24]. In neurosurgery, assessing HRQoL and disease-specific symptoms is essential in the field of skull base tumors, as the long-term outcome of these usually benign lesions with potential involvement of neurovascular structures has to be considered in treatment decisions [15,25,26]. The complementary effect of using both generic and specific questionnaires needs to be emphasized; however, reports on the application of disease-specific questionnaires in skull base tumors are scarce [27,28]. The current study presents the first experiences and pitfalls of an elaborate strategy to assess HRQoL using digital PROMs in a tertiary care center specialized in skull base diseases, following a sophisticated protocol including validated and well-established, both generic and disease-specific questionnaires.

### 4.1. Availability of PROMs and Participation Rate

The inclusion of individual patient-specific factors in decision-making processes requires the compliance of those affected to obtain representative results. It is generally distinguished between *personal characteristics of non-participants* and *individual reasons for non-participation* [29]. The literature is scarce regarding descriptions of personal characteristics of non-participants, although two studies observed an emphasis on female gender [29,30,31]. In contrast, numerous reasons for non-participation are reported that reflect the results of our study [30]. For example, studies listed “health problems” and “old age” as reasons for non-participation, as they impeded participation due to problems with handling or cognition [30,32,33,34]. These problems were also identified in our data, which were labeled “cognitive impairment/age/lack of vision”. Beside “language barrier” as another reason, “lack of engagement” is also found in the literature, suggesting that patients feel “not sick enough” to participate or do not see a personal benefit [20,30,31,35,36]. “Lack of engagement” might have been a factor in our study when patients did not participate for “unknown reasons”. Inconsistent staff availability with inconsistent ability to implement PROMs may also have affected personal engagement and, thus, influenced the decision not to participate. As “personal reasons”, factors such as “data security and trust” (i.e., concerns about the digital format), “technical issues” (lack of hardware/software or lack of technical skills), and “emotional distress” (e.g., patients do not want to be reminded of their illness by follow-up interviews) can be found both in the literature and in our analysis [30,35,37,38,39,40,41].

### 4.2. Conduction of PROMs in Relation to the COVID-19 Pandemic

During COVID-19 pandemic peaks, the demand for specialized neurosurgical consultations remained high, as demonstrated by our analysis over 2 years. Comparing the 13 months covered by COVID-19 waves and the 11 months beyond, there were no relevant differences regarding the capacity of medical staff to implement PROMs or (what depends on it) the mean number of completed PROMs per SBCD. However, there were significant differences when comparing these aspects between the first and second year after implementation of PROMs, independent from COVID-19 waves. The number of SBCDs where PROMs could not be implemented due to staff unavailability was twice as high in the second year compared with the first year; consequently, the mean number of PROMs completed per SBCD was significantly reduced in the second year. This implies a certain bias in data collection, as the implementation of PROMs was thus significantly influenced by the availability of personnel. It can be assumed that more PROMs could have been implemented if staff had been available, and that many people who would have wished to participate did not have the opportunity to do so. Therefore, the availability of medical staff is a crucial factor for initiating PROMs. As this aspect has been identified as a key factor based on our data, intensive efforts should be made to find solutions for the future. If it is not possible to provide more medical staff, scientific staff or students could be involved to support the work, with the aim of consistent data collection and provision for individualized medical care. Several reports published in 2022 pointed to a progressive decline in medical staff availability in the aftermath of the COVID-19 pandemic [42,43]. In addition to the immediate negative effects on maintaining patient care, also long-term adverse effects on future healthcare quality are to be expected.

### 4.3. Response Rate to PROMs Depending on Subgroup and Neurosurgical Procedure

The current analysis revealed a response rate of 52.5% at 3-month follow-up and of 38.6% at 12-month follow-up. Few similar studies on the use of PROMs in skull base diseases can be found in the literature, and no comparable data on response rates are available. Several reports describe the assessment of the general HRQoL or the development of specific questionnaires for application in certain skull base diseases [6,7,14,44]. A review by Nielsen et al. of 51 studies reported high variance in dropout rates regarding the general application of PROMs in medicine, where a high dependency on the underlying disease and the conducted treatment can be assumed [29]. Most patients who completed T1 were affected by benign tumors and were equally distributed among the subgroups AMSB (43.7%) and CPA (43.0%). There were no relevant differences in response rates during follow-up between these subgroups. Thus, it was neither observed that response rates depended on specific diseases or (localization-related) neurological deficits, nor, for example, that the higher amount of specific questionnaires (PANQOL, DHI, FDI, and FaCE) in subgroup CPA had a negative “lack of engagement”effect on compliance in completing follow-up. 

Among 158 patients, 24.7% underwent surgery between T1 and T2, 37.3% followed a wait-and-scan strategy, and 38.0% had already undergone surgery before T1. Patients who underwent surgery after T1 had the highest response rates during follow-up (T2: 64.1% and T3: 53.8%), assuming that patients’ motivation in the immediate context of treatment is high and that recent surgery as a major event is strongly present in their mind. This might create a selection bias for patients who underwent surgery between T1 and T2, which could lead to an overall overrepresentation of this group. However, in the context of recent surgery, there are also certain conditions that may prevent participation or influence the results, such as complications requiring additional treatment, re-admission to hospital, or mortality. These circumstances are of special importance for the interpretation of the results of questionnaires. At 3-month follow-up, the response rate among patients following a wait-and-scan strategy (52.5%) was slightly higher than that among patients having undergone surgery before T1 (45.0%). At 12-month follow-up, patients following a wait-and-scan strategy had lower response rates (28.8%) compared with those having undergone surgery before T1 (38.3%). This might be attributed to the effect described in the literature, where patients affected by asymptomatic “incidental findings” may have less mental focus on the disease, feel “not sick enough”, or see less personal benefit from participating [30,31,35]. In contrast, after having undergone surgery as a “life event”, a disease may be more mentally present in the long-term, resulting in higher motivation to attend follow-up assessments. The knowledge of patient-specific factors appears to be essential in wait-and-scan strategies, where decision making about a need for treatment must be repeated regularly. Therefore, the importance of performing follow-up PROMs should be emphasized in patients following wait-and-scan strategies. As a consequence of our analysis, efforts should be made to provide additional staff resources to regularly monitor response rates and, in case of non-response, to try to increase response rates through personal contact and motivation.

### 4.4. Response Rate to PROMs Depending on Age

The present analysis revealed a significantly lower mean age of patients who completed T3 compared with the total number of patients who completed T1 and those who did not complete T3. Thus, there appears a correlation between older age and a lower response rate during follow-up, which is also described in the literature [30,32,33,34]. This also suggests an association with the digital format, as older people are more likely to lack technical tools and skills. Consequently, there might be a risk for older people to be underrepresented by the application of a digital format. Family members or medical staff as support for conducting digital PROMs in elderly people and those with cognitive/visual impairment or other neurological deficits could help to avert the risk of disadvantaging vulnerable groups. As already indicated above, the involvement of scientific staff or students could be helpful for the conduction in these groups. Furthermore, providing questionnaires in different languages seems to be important in this context and should be feasible, especially in digital formats with automated evaluation.

### 4.5. Response Rate to PROMs Depending on Gender

During follow-up, the response rates of male participants were slightly higher than those of female participants, which may be in line with the observation reported in the literature that non-participants were more likely to be female [29,30,31]. However, no critical differences necessitating gender-specific interventions for improvement were identified.

## 5. Conclusions

The implementation of digital PROMs for the assessment of HRQoL may provide perspectives in the trend toward individualized patient care, also in the context of (mostly benign) skull base diseases. The current evaluation of our strategy to conduct digital PROMs revealed high response rates during follow-up, with no significant differences regarding gender, and a trend for best response rates associated with recent surgery. Older age was frequently a reason for non-participation and was a significant risk factor for not completing follow-up assessments. The lack of medical personnel was found to be the key problem preventing a widespread implementation and supervision of digital PROMs, particularly for enhanced disease-specific protocols. Reduced staff capacity in our analysis was less of an acute problem during the COVID-19 waves, but rather became apparent as a long-term effect of the pandemic.

## Figures and Tables

**Table 1:** Evaluation of the response rate to PROMs depending on subgroup and neurosurgical procedure.

		In Total	Subgroups		
		ALL	AMSB	CPA	NVC
**Total no. of Patients with Completed T1 (n)**		158	69	68	21
**no. of patients with completed T1 per procedure** (n, % in relation to no. of T1 of the corresponding group)	surgery between T1 and T2	39 (24.7%)	16 (23.2%)	18 (26.5%)	5 (23.8%)
	wait-and-scan strategy	59 (37.3%)	24 (34.8%)	24 (35.3%)	11 (52.4%)
	previous surgery before T1 ^§^	60 (38.0%)	29 (42.0%)	26 (38.2%)	5 (23.8%)
**total no. of patients with completed T2 (n)**		83	36	41 ^&^	6 ^&&^
**no. of patients with completed T2 per procedure** (n, % in relation to no. of T2 of the corresponding group)	surgery between T1 and T2	25 (30.1%)	10 (27.8%)	13 (31.7%)	2 (33.3%)
	wait-and-scan strategy	31 (37.4%)	11 (30.5%)	16 (39.0%)	4 (66.7%)
	previous surgery before T1	27 (32.5%)	15 (41.7%)	12 (29.3%)	0 (0.0%)
**total no. of patients with completed T3 (n)**		61	27 ^#^	26 ^##^	8 ^###^
**no. of patients with completed T3 per procedure** (n, % in relation to no. of T3 of the corresponding group)	surgery between T1 and T2	21 (34.4%)	9 (33.3%)	9 (34.6%)	3 (37.5%)
	wait-and-scan strategy	17 (27.9%)	7 (25.9%)	6 (23.1%)	4 (50.0%)
	previous surgery before T1	23 (37.7%)	11 (40.8%)	11 (42.3%)	1 (12.5%)

ALL = total group; AMSB = subgroup “anterior and middle skull base”; CPA = subgroup “cerebellopontine angle”; NVC = subgroup “neurovascular conflict”; no. = number; n = number of completed questionnaires; T1 = initial assessment; T2 = follow-up assessment after 3 months; T3 = follow-up assessment after 12 months; “surgery between T1 and T2” = surgical treatment was performed after the initial assessment and before the follow-up assessment after 3 months; “previous surgery before T1” = surgical treatment had already been performed before the initial assessment; ^§^ = in 10/29 (34.5%) patients in subgroup AMSB and in 9/26 (34.6%) patients in subgroup CPA, surgery had been performed within 3 to 5 months before T1; ^&–&&^ = in 1 patient in subgroup CPA (^&^) and in 2 patients in subgroup NVC (^&&^), the time for T2 was not reached within the analysis period; ^#–###^ = in 9 patients in subgroup AMSB (^#^), in 15 patients in subgroup CPA (^##^), and in 3 patients in subgroup NVC (^###^), the time for T3 was not reached within the analysis period.

## Data Availability

The data used to support the findings of this study are available from the corresponding author upon request.
